# Crystal structure and Hirshfeld surface analysis of 2-amino-4-meth­oxy-6-methyl­pyrimidinium 2-hy­droxy­benzoate

**DOI:** 10.1107/S2056989017011252

**Published:** 2017-08-08

**Authors:** Muthaiah Jeevaraj, Palaniyappan Sivajeyanthi, Bellarmin Edison, Kaliyaperumal Thanigaimani, Kasthuri Balasubramani, Ibrahim Abdul Razak

**Affiliations:** a Department of Chemistry, Government Arts College (Autonomous), Thanthonimalai, Karur 639 005, Tamil Nadu, India; b Department of Chemistry, Government Arts College, Tiruchirappalli 620 022, Tamil Nadu, India; c School of Physics, Universiti Sains Malaysia, 11800 USM, Penang, Malaysia

**Keywords:** crystal structure, Hirshfeld surface analysis, hydrogen bonding

## Abstract

In the title mol­ecular salt, C_6_H_10_N_3_O^+^·C_7_H_5_O_3_^−^, the cation is protonated at the N atom lying between the amine and methyl substituents and the dihedral angle between the carboxyl group and its attached ring in the anion is 4.0 (2)°. The anion features an intra­molecular O—H⋯O hydrogen bond, which closes an *S*(6) ring. The cation and anion are linked by two N—H⋯O hydrogen bonds [*R*_2_^2^(8) motif] to generate an ion pair in which the dihedral angle between the aromatic rings is 8.34 (9)°. Crystal symmetry relates two ion pairs bridged by further N—H⋯O hydrogen bonds into a tetra­meric *DDAA* array. The tetra­mers are linked by pairs of C—H⋯O hydrogen bonds to generate [100] chains. Hirshfeld surface and fingerprint plot analyses are presented.

## Abstract 2

Tetra­meric associations of two cations and two anions occur, being linked by N—H⋯O hydrogen bonds.

## chemical context   

Pyrimidine and amino­pyrimidine derivatives have many applications as pesticides and pharmaceutical agents (Condon *et al.*, 1993 ▸). For example, imazosulfuron, ethirmol and mepanipyrim have been commercialized as agrochemicals (Maeno *et al.*, 1990 ▸). Pyrimidine derivatives have also been developed as anti­viral agents, such as AZT, which is the most widely-used anti-AIDS drug (Gilchrist, 1997 ▸). Hydrogen bonding plays a vital role in mol­ecular recognition. Supra­molecular chemistry plays a pivotal role in biological systems and in artificial systems. It refers to the specific inter­action between two or more motifs through non-covalent inter­actions such as hydrogen bonding, hydro­phobic forces, van der Waals forces, π–π inter­actions *etc*. The generating of supra­molecular architectures is correlated to the positions and properties of the active groups in mol­ecules (Desiraju *et al.*, 1989 ▸; Steiner *et al.*, 2002 ▸) As part of our studies in these areas, the synthesis and structure of the title mol­ecular salt, (I), is presented here.
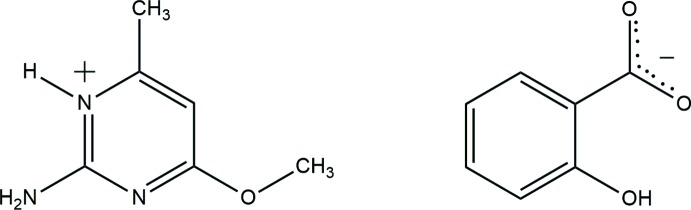


## structural commentary   

**Figure 1:**
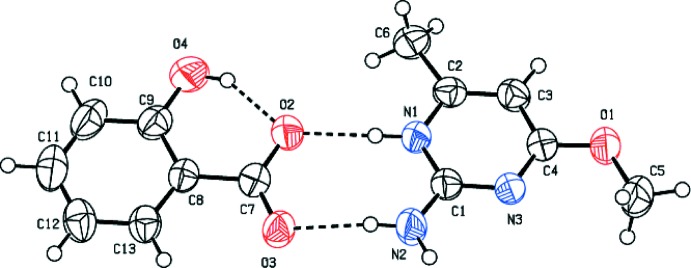
The asymmetric unit of (I), with 50% probability displacement ellipsoids. The hydrogen bonds are indicated by dashed lines.

The mol­ecular structure of (I) is shown in Fig. 1 ▸. The asymmetric unit contains a 2-amino-4-meth­oxy-6-methyl­pyrimidinium cation and a 2-hy­droxy­benzoate anion. The cation is protonated at N1, which lies between the amine and methyl substituents: this protonation is reflected by an increase in the bond angle at N1 [C1—N1—C2 = 121.09 (15)°], when compared with the unprotonated atom N3 [C1—N3—C4 = 116.52 (18)°], and the corresponding angle of 116.01 (18)° in neutral 2-amino-4-meth­oxy-6-methyl­pyrimidine (Glidewell *et al.*, 2003 ▸). An intra­molecular O—H⋯O hydrogen bond occurs within the anion (Table 1 ▸).

## supra­molecular features   

**Figure 2:**
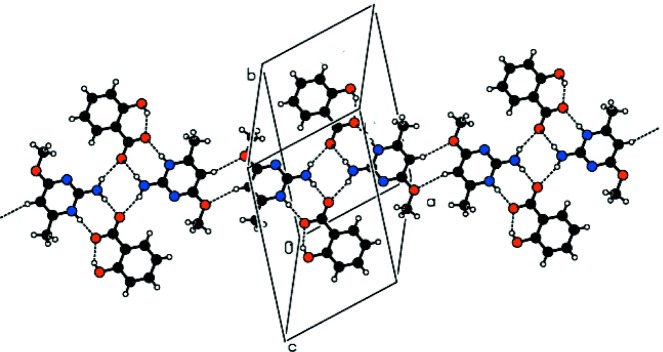
A [100] chain in the crystal of (I) incorporating 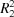
(8), 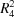
(8) and 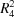
(12) ring motifs.

The protonated N atom (N1) and 2-amino group (N2) of the cation inter­acts with the O1 and O2 oxygen atoms of the carboxyl­ate anion through a pair of N—H⋯O hydrogen bonds (Table 1 ▸), forming an eight-membered ring motif 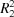
(8). Inversion-related 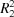
(8) ring motifs are further bridged by N—H⋯O hydrogen bonds thereby forming a *DDAA* tetra­mer (*D* stands for hydrogen-bond donor and *A* stands for hydrogen-bond acceptor). This set of fused rings can be represented by the graph-set notations 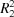
(8), 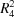
(8) and 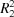
(8). This type of motif has been reported previously in the crystal structures of trimethoprim hydrogen glutarate (Robert *et al.*, 2001 ▸) and 2-amino-4-meth­oxy-6-methyl­pyridinium tri­fluoro­acetate (Jeevaraj *et al.*, 2016 ▸). These arrays are further linked *via* pairwise C—H⋯O hydrogen bonds to generate another 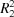
(8) ring motif as part of a [100] chain (Fig. 2 ▸).

## Hirshfeld surface analysis   

**Figure 3:**
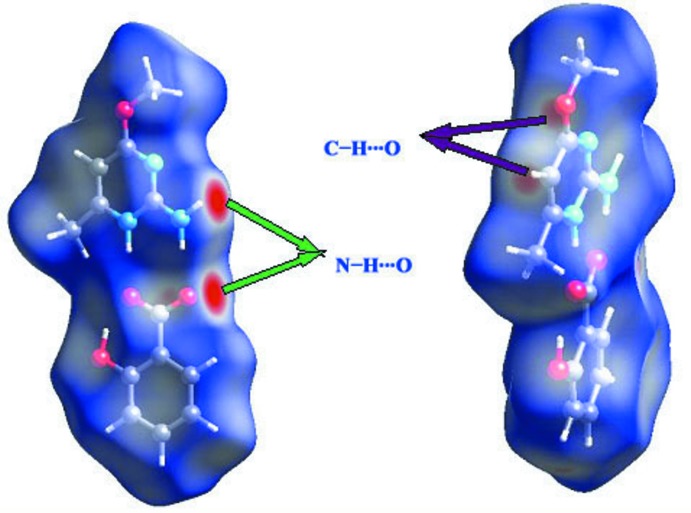
Three-dimensional Hirshfeld surface of (I).

**Figure 4:**
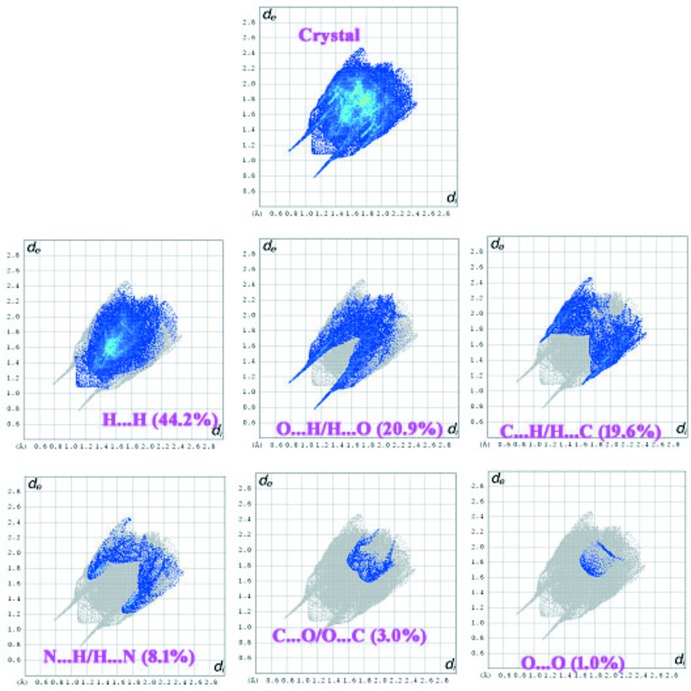
Fingerprint plots for (I).

The *d*_norm_ parameter takes negative or positive values depending on whether the inter-mol­ecular contact is shorter or longer, respectively, than the van der Waals radii (Spackman & Jayatilaka *et al.*, 2009 ▸; McKinnon *et al.*, 2007 ▸). The *d*_norm_ surface of the ion-pair in (I) is shown in Fig. 3 ▸: this naturally neglects hydrogen bonds (intra-anion O—H⋯O and N—H⋯O cation-to-anion) that occur within the asymmetric unit. The red points represent closer contacts and negative *d*_norm_ values on the surface corresponding to the N—H⋯O and C—H⋯O inter­actions are light red in colour. Two-dimensional fingerprint plots from the Hirshfeld surface analysis, as shown in Fig. 4 ▸, give a break-down of different contacts as follows: H⋯H (44.2%), C⋯H/H⋯C (19.6%), O⋯H/H⋯O (20.9%), C⋯O/O⋯C (3.0%), C⋯C (2.9%), N⋯H/H⋯N (8.1%) and O⋯O (1.0%). Two ‘wingtips’ in the fingerprint plot are related to the strong H⋯O and O⋯H inter­actions.

## Database survey   

A search of the Cambridge Structural Database (Version 5.37, update February 2017; Groom *et al.*, 2016 ▸) for 2-amino-4-meth­oxy-6-methyl­pyrimidine yielded seven structures: VAQSOW, VAQSUC, VAQSEM, VAQSIQ, VAQRUB and VAQSAI (Aakeroy *et al.*, 2003 ▸) and NUQTOJ (Jasinski *et al.* (2010 ▸).

## Synthesis and crystallization   

The title compound was synthesized by mixing hot methano­lic solutions (20 ml) of 2-amino-4-meth­oxy-6-methyl­pyrimidine (0.139 mg) and 2-hy­droxy­benzoic acid (0.156 mg) in a 1:1 molar ratio. The mixed solutions were warmed few minutes over a waterbath and then cooled and kept at room temperature for slow evaporation. After a few days, colourless block-shaped crystals of (I) were obtained (yield = 65%).

## Refinement   

Crystal data, data collection and structure refinement details are summarized in Table 2 ▸. The hydrogen atoms were positioned geometrically (N—H = 0.86, O—H = 0.82 and C—H = 0.96 or 0.93 Å) and were refined using a riding model, with *U*_iso_(H) = 1.2*U*_eq_(C) or 1.5*U*_eq_(methyl C). A rotating-group model was used for the methyl group.

## Supplementary Material

Crystal structure: contains datablock(s) global, I, 1. DOI: 10.1107/S2056989017011252/hb7693sup1.cif

Structure factors: contains datablock(s) I. DOI: 10.1107/S2056989017011252/hb7693Isup2.hkl

Supporting information file. DOI: 10.1107/S2056989017011252/hb7693Isup3.cml

CCDC reference: 1559280

Additional supporting information:  crystallographic information; 3D view; checkCIF report

## Figures and Tables

**Table 1:** Hydrogen-bond geometry (Å, °)

*D*—H⋯*A*	*D*—H	H⋯*A*	*D*⋯*A*	*D*—H⋯*A*
N1—H1⋯O2	0.86	1.84	2.7033 (19)	176
N2—H2*A*⋯O3^i^	0.86	2.00	2.816 (3)	158
N2—H2*B*⋯O3	0.86	1.99	2.830 (2)	165
O4—H4⋯O2	0.82	1.81	2.534 (2)	147
C3—H3⋯O1^ii^	0.93	2.48	3.374 (3)	160

Symmetry codes: (i) 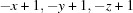; (ii) 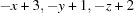.

**Table 2:** Experimental details

Crystal data	
Chemical formula	C_6_H_10_N_3_O^+^·C_7_H_5_O_3_^−^
*M*_r_	277.28
Crystal system, space group	Monoclinic, *P*2_1_/*c*
Temperature (K)	296
*a*, *b*, *c* (Å)	9.4291 (12), 15.0620 (19), 12.1595 (11)
β (°)	128.252 (6)
*V* (Å^3^)	1356.1 (3)
*Z*	4
Radiation type	Mo *K*α
μ (mm^−1^)	0.10
Crystal size (mm)	0.55 × 0.33 × 0.16

Data collection	
Diffractometer	Bruker KappaCCD APEXII
Absorption correction	Multi-scan (*SADABS*; Bruker, 2004 ▸)
*T*_min_, *T*_max_	0.960, 0.984
No. of measured, independent and observed [*I* > 2σ(*I*)] reflections	33325, 4033, 2373
*R*_int_	0.042
(sin θ/λ)_max_ (Å^−1^)	0.708

Refinement	
*R*[*F* ^2^ > 2σ(*F* ^2^)], *wR*(*F* ^2^), *S*	0.056, 0.164, 1.02
No. of reflections	4033
No. of parameters	183
H-atom treatment	H atoms treated by a mixture of independent and constrained refinement
Δρ_max_, Δρ_min_ (e Å^−3^)	0.21, −0.19

Computer programs: *APEX2* and *SAINT* (Bruker, 2004 ▸), *SHELXTL* (Sheldrick, 2008 ▸) and *PLATON* (Spek, 2009 ▸).

## supplementary crystallographic information

### Crystal data

**Table:**

C_6_H_10_N_3_O^+^·C_7_H_5_O_3_^−^	*F*(000) = 584
*M**_r_* = 277.28	*D*_x_ = 1.358 Mg m^−^^3^
Monoclinic, *P*2_1_/*c*	Mo *K*α radiation, λ = 0.71073 Å
Hall symbol: -P 2ybc	Cell parameters from 4033 reflections
*a* = 9.4291 (12) Å	θ = 2.5–27.5°
*b* = 15.0620 (19) Å	µ = 0.10 mm^−^^1^
*c* = 12.1595 (11) Å	*T* = 296 K
β = 128.252 (6)°	Block, colourless
*V* = 1356.1 (3) Å^3^	0.55 × 0.33 × 0.16 mm
*Z* = 4	

### Data collection

**Table:**

Bruker KappaCCD APEXII diffractometer	4033 independent reflections
Radiation source: fine-focus sealed tube	2373 reflections with *I* > 2σ(*I*)
Graphite monochromator	*R*_int_ = 0.042
ω and φ scans	θ_max_ = 30.2°, θ_min_ = 2.5°
Absorption correction: multi-scan (SADABS; Bruker, 2004)	*h* = −13→13
*T*_min_ = 0.960, *T*_max_ = 0.984	*k* = −21→21
33325 measured reflections	*l* = −17→17

### Refinement

**Table:**

Refinement on *F*^2^	Primary atom site location: structure-invariant direct methods
Least-squares matrix: full	Secondary atom site location: difference Fourier map
*R*[*F*^2^ > 2σ(*F*^2^)] = 0.056	Hydrogen site location: inferred from neighbouring sites
*wR*(*F*^2^) = 0.164	H atoms treated by a mixture of independent and constrained refinement
*S* = 1.02	*w* = 1/[σ^2^(*F*_o_^2^) + (0.0587*P*)^2^ + 0.4004*P*] where *P* = (*F*_o_^2^ + 2*F*_c_^2^)/3
4033 reflections	(Δ/σ)_max_ < 0.001
183 parameters	Δρ_max_ = 0.21 e Å^−^^3^
0 restraints	Δρ_min_ = −0.19 e Å^−^^3^

### Special details

**Table:**

Geometry. Bond distances, angles etc. have been calculated using the rounded fractional coordinates. All su's are estimated from the variances of the (full) variance-covariance matrix. The cell esds are taken into account in the estimation of distances, angles and torsion angles
Refinement. Refinement of F^2^ against ALL reflections. The weighted R-factor wR and goodness of fit S are based on F^2^, conventional R-factors R are based on F, with F set to zero for negative F^2^. The threshold expression of F^2^ > 2sigma(F^2^) is used only for calculating R-factors(gt) etc. and is not relevant to the choice of reflections for refinement. R-factors based on F^2^ are statistically about twice as large as those based on F, and R- factors based on ALL data will be even larger.

### Fractional atomic coordinates and isotropic or equivalent isotropic displacement parameters (Å^2^)

**Table:**

	*x*	*y*	*z*	*U*_iso_*/*U*_eq_	
O1	1.29929 (16)	0.42997 (10)	1.00372 (13)	0.0632 (4)	
N1	0.97381 (17)	0.55436 (9)	0.63943 (14)	0.0475 (4)	
N2	0.72932 (19)	0.50804 (12)	0.62236 (17)	0.0659 (6)	
N3	1.01155 (18)	0.46656 (10)	0.81604 (14)	0.0486 (4)	
C1	0.9058 (2)	0.50922 (12)	0.69373 (17)	0.0482 (5)	
C2	1.1545 (2)	0.55729 (11)	0.70701 (17)	0.0465 (5)	
C3	1.2648 (2)	0.51464 (12)	0.82987 (18)	0.0510 (5)	
C4	1.1868 (2)	0.46995 (11)	0.88151 (17)	0.0472 (5)	
C5	1.2254 (3)	0.38111 (18)	1.0601 (3)	0.0863 (9)	
C6	1.2148 (3)	0.60934 (14)	0.6387 (2)	0.0620 (7)	
O2	0.75239 (17)	0.63571 (9)	0.38790 (13)	0.0593 (4)	
O3	0.51173 (16)	0.57024 (9)	0.34276 (13)	0.0597 (4)	
O4	0.7259 (2)	0.72936 (11)	0.20277 (15)	0.0756 (6)	
C7	0.5836 (2)	0.62036 (11)	0.30856 (17)	0.0459 (5)	
C8	0.4695 (2)	0.66507 (10)	0.16945 (16)	0.0445 (5)	
C9	0.5463 (3)	0.71779 (12)	0.12434 (19)	0.0554 (6)	
C10	0.4349 (4)	0.75988 (14)	−0.0047 (2)	0.0723 (8)	
C11	0.2513 (3)	0.74946 (14)	−0.0879 (2)	0.0719 (8)	
C12	0.1737 (3)	0.69718 (14)	−0.0458 (2)	0.0649 (7)	
C13	0.2822 (2)	0.65538 (12)	0.08200 (19)	0.0529 (6)	
H1	0.90150	0.58160	0.56090	0.0570*	
H2A	0.68270	0.47990	0.65460	0.0790*	
H2B	0.66070	0.53540	0.54350	0.0790*	
H3	1.38930	0.51480	0.87920	0.0610*	
H5A	1.14960	0.41960	1.06650	0.1290*	
H5B	1.32220	0.35930	1.15160	0.1290*	
H5C	1.15550	0.33200	0.99970	0.1290*	
H6A	1.22890	0.67050	0.66590	0.0930*	
H6B	1.12630	0.60460	0.53890	0.0930*	
H6C	1.32820	0.58650	0.66740	0.0930*	
H4	0.77590	0.70480	0.27870	0.1130*	
H10	0.48530	0.79540	−0.03500	0.0870*	
H11	0.17840	0.77820	−0.17400	0.0860*	
H12	0.04900	0.69010	−0.10300	0.0780*	
H13	0.22980	0.61990	0.11070	0.0640*	

### Atomic displacement parameters (Å^2^)

**Table:**

	*U*^11^	*U*^22^	*U*^33^	*U*^12^	*U*^13^	*U*^23^
O1	0.0472 (7)	0.0777 (9)	0.0528 (7)	0.0025 (6)	0.0250 (6)	0.0194 (6)
N1	0.0436 (7)	0.0531 (8)	0.0423 (7)	−0.0014 (6)	0.0249 (6)	0.0067 (6)
N2	0.0418 (8)	0.0915 (12)	0.0561 (9)	0.0001 (8)	0.0262 (7)	0.0254 (9)
N3	0.0426 (7)	0.0549 (8)	0.0442 (8)	−0.0032 (6)	0.0249 (6)	0.0054 (6)
C1	0.0433 (8)	0.0543 (10)	0.0446 (9)	−0.0026 (7)	0.0260 (8)	0.0036 (7)
C2	0.0468 (9)	0.0486 (9)	0.0478 (9)	−0.0061 (7)	0.0311 (8)	−0.0041 (7)
C3	0.0417 (8)	0.0597 (10)	0.0492 (9)	−0.0022 (7)	0.0269 (8)	0.0016 (8)
C4	0.0447 (9)	0.0496 (9)	0.0428 (9)	0.0003 (7)	0.0248 (7)	0.0021 (7)
C5	0.0666 (13)	0.1089 (19)	0.0698 (14)	−0.0019 (12)	0.0355 (12)	0.0374 (13)
C6	0.0594 (11)	0.0715 (12)	0.0613 (11)	−0.0109 (9)	0.0405 (10)	0.0042 (9)
O2	0.0508 (7)	0.0703 (8)	0.0485 (7)	−0.0047 (6)	0.0266 (6)	0.0078 (6)
O3	0.0537 (7)	0.0726 (9)	0.0564 (7)	0.0029 (6)	0.0359 (6)	0.0161 (6)
O4	0.0751 (9)	0.0866 (11)	0.0666 (9)	−0.0209 (8)	0.0446 (8)	0.0052 (8)
C7	0.0511 (9)	0.0460 (9)	0.0460 (9)	0.0039 (7)	0.0328 (8)	0.0010 (7)
C8	0.0540 (9)	0.0387 (8)	0.0421 (8)	0.0046 (7)	0.0304 (8)	0.0002 (6)
C9	0.0703 (12)	0.0485 (9)	0.0518 (10)	−0.0050 (8)	0.0400 (10)	−0.0011 (8)
C10	0.1055 (18)	0.0579 (12)	0.0625 (13)	−0.0020 (11)	0.0565 (13)	0.0097 (10)
C11	0.0920 (16)	0.0589 (12)	0.0491 (11)	0.0191 (11)	0.0359 (12)	0.0094 (9)
C12	0.0653 (12)	0.0627 (12)	0.0528 (11)	0.0186 (9)	0.0296 (10)	0.0042 (9)
C13	0.0571 (10)	0.0490 (10)	0.0502 (10)	0.0100 (8)	0.0320 (9)	0.0026 (8)

### Geometric parameters (Å, º)

**Table:**

O1—C4	1.321 (2)	C5—H5A	0.9600
O1—C5	1.445 (4)	C5—H5C	0.9600
O2—C7	1.271 (3)	C5—H5B	0.9600
O3—C7	1.245 (3)	C6—H6B	0.9600
O4—C9	1.345 (3)	C6—H6C	0.9600
O4—H4	0.8200	C6—H6A	0.9600
N1—C2	1.359 (3)	C7—C8	1.490 (2)
N1—C1	1.354 (3)	C8—C13	1.395 (3)
N2—C1	1.317 (3)	C8—C9	1.396 (3)
N3—C1	1.335 (2)	C9—C10	1.388 (3)
N3—C4	1.318 (3)	C10—C11	1.370 (4)
N1—H1	0.8600	C11—C12	1.372 (4)
N2—H2B	0.8600	C12—C13	1.375 (3)
N2—H2A	0.8600	C10—H10	0.9300
C2—C6	1.488 (3)	C11—H11	0.9300
C2—C3	1.343 (2)	C12—H12	0.9300
C3—C4	1.400 (3)	C13—H13	0.9300
C3—H3	0.9300		
			
O1···C3^i^	3.374 (3)	C7···H10^iv^	2.8800
O2···C3^ii^	3.411 (2)	C7···H2B	2.7800
O2···N1	2.7033 (19)	C7···H1	2.7100
O2···O4	2.534 (2)	C7···H4	2.4200
O3···N2^iii^	2.816 (3)	C9···H5B^vi^	3.0500
O3···N2	2.830 (2)	C12···H6A^ix^	3.0300
O4···O2	2.534 (2)	C13···H6A^ix^	2.9700
O1···H3^i^	2.4800	H1···O2	1.8400
O2···H4	1.8100	H1···O3	2.9200
O2···H6B	2.8300	H1···C7	2.7100
O2···H1	1.8400	H1···H2B	2.2600
O3···H2B	1.9900	H1···H6B	2.3200
O3···H13	2.5100	H2A···O3^iii^	2.0000
O3···H1	2.9200	H2A···H2B^iii^	2.5800
O3···H2A^iii^	2.0000	H2B···O3	1.9900
O3···H6C^ii^	2.8500	H2B···C7	2.7800
O3···H10^iv^	2.6100	H2B···H1	2.2600
O4···H12^v^	2.7200	H2B···H2A^iii^	2.5800
O4···H5B^vi^	2.8600	H3···H6C	2.5100
N1···O2	2.7033 (19)	H3···O1^i^	2.4800
N2···O3	2.830 (2)	H3···C3^i^	3.0100
N2···O3^iii^	2.816 (3)	H3···H3^i^	2.3600
N3···H5C	2.6800	H4···O2	1.8100
N3···H5A	2.5600	H4···C7	2.4200
C2···C11^vii^	3.551 (3)	H4···H12^v^	2.5700
C2···C12^vii^	3.582 (3)	H5A···N3	2.5600
C3···O1^i^	3.374 (3)	H5B···O4^x^	2.8600
C3···C12^vii^	3.492 (3)	H5B···C9^x^	3.0500
C3···C7^ii^	3.463 (3)	H5C···N3	2.6800
C3···O2^ii^	3.411 (2)	H6A···C13^v^	2.9700
C4···C12^vii^	3.556 (3)	H6A···C12^v^	3.0300
C4···C13^vii^	3.441 (3)	H6B···O2	2.8300
C6···C12^v^	3.550 (3)	H6B···H1	2.3200
C7···C3^ii^	3.463 (3)	H6C···H3	2.5100
C11···C2^viii^	3.551 (3)	H6C···O3^ii^	2.8500
C12···C3^viii^	3.492 (3)	H10···O3^xi^	2.6100
C12···C2^viii^	3.582 (3)	H10···C7^xi^	2.8800
C12···C4^viii^	3.556 (3)	H12···O4^ix^	2.7200
C12···C6^ix^	3.550 (3)	H12···H4^ix^	2.5700
C13···C4^viii^	3.441 (3)	H13···O3	2.5100
C3···H3^i^	3.0100		
			
C4—O1—C5	118.6 (2)	C2—C6—H6A	109.00
C9—O4—H4	109.00	C2—C6—H6C	109.00
C1—N1—C2	121.09 (15)	H6A—C6—H6B	109.00
C1—N3—C4	116.52 (18)	C2—C6—H6B	109.00
C1—N1—H1	119.00	H6B—C6—H6C	110.00
C2—N1—H1	119.00	H6A—C6—H6C	109.00
H2A—N2—H2B	120.00	O2—C7—C8	117.66 (18)
C1—N2—H2A	120.00	O3—C7—C8	119.57 (18)
C1—N2—H2B	120.00	O2—C7—O3	122.77 (16)
N2—C1—N3	119.68 (19)	C7—C8—C13	120.12 (18)
N1—C1—N3	122.16 (19)	C9—C8—C13	118.69 (16)
N1—C1—N2	118.17 (16)	C7—C8—C9	121.19 (19)
N1—C2—C6	116.68 (16)	O4—C9—C10	118.8 (3)
C3—C2—C6	125.0 (2)	C8—C9—C10	119.4 (3)
N1—C2—C3	118.35 (19)	O4—C9—C8	121.81 (17)
C2—C3—C4	118.0 (2)	C9—C10—C11	120.6 (3)
N3—C4—C3	123.91 (16)	C10—C11—C12	120.8 (2)
O1—C4—C3	116.42 (19)	C11—C12—C13	119.3 (2)
O1—C4—N3	119.67 (18)	C8—C13—C12	121.3 (2)
C2—C3—H3	121.00	C9—C10—H10	120.00
C4—C3—H3	121.00	C11—C10—H10	120.00
O1—C5—H5B	109.00	C10—C11—H11	120.00
O1—C5—H5C	109.00	C12—C11—H11	120.00
O1—C5—H5A	109.00	C11—C12—H12	120.00
H5A—C5—H5C	110.00	C13—C12—H12	120.00
H5B—C5—H5C	109.00	C8—C13—H13	119.00
H5A—C5—H5B	110.00	C12—C13—H13	119.00
			
C5—O1—C4—N3	1.9 (3)	O2—C7—C8—C13	−175.72 (17)
C5—O1—C4—C3	−178.55 (18)	O3—C7—C8—C9	−176.44 (18)
C2—N1—C1—N2	−179.22 (17)	O3—C7—C8—C13	4.1 (3)
C2—N1—C1—N3	0.8 (3)	C7—C8—C9—O4	1.2 (3)
C1—N1—C2—C3	−0.4 (2)	C7—C8—C9—C10	−178.58 (19)
C1—N1—C2—C6	−179.52 (16)	C13—C8—C9—O4	−179.40 (18)
C4—N3—C1—N1	−0.5 (3)	C13—C8—C9—C10	0.8 (3)
C4—N3—C1—N2	179.56 (17)	C7—C8—C13—C12	178.86 (18)
C1—N3—C4—O1	179.29 (16)	C9—C8—C13—C12	−0.6 (3)
C1—N3—C4—C3	−0.3 (3)	O4—C9—C10—C11	179.7 (2)
N1—C2—C3—C4	−0.3 (3)	C8—C9—C10—C11	−0.5 (3)
C6—C2—C3—C4	178.73 (18)	C9—C10—C11—C12	−0.2 (4)
C2—C3—C4—O1	−178.88 (16)	C10—C11—C12—C13	0.4 (4)
C2—C3—C4—N3	0.7 (3)	C11—C12—C13—C8	−0.1 (3)
O2—C7—C8—C9	3.7 (3)		

Symmetry codes: (i) −*x*+3, −*y*+1, −*z*+2; (ii) −*x*+2, −*y*+1, −*z*+1; (iii) −*x*+1, −*y*+1, −*z*+1; (iv) *x*, −*y*+3/2, *z*+1/2; (v) *x*+1, −*y*+3/2, *z*+1/2; (vi) −*x*+2, *y*+1/2, −*z*+3/2; (vii) *x*+1, *y*, *z*+1; (viii) *x*−1, *y*, *z*−1; (ix) *x*−1, −*y*+3/2, *z*−1/2; (x) −*x*+2, *y*−1/2, −*z*+3/2; (xi) *x*, −*y*+3/2, *z*−1/2.

### Hydrogen-bond geometry (Å, º)

**Table:**

*D*—H···*A*	*D*—H	H···*A*	*D*···*A*	*D*—H···*A*
N1—H1···O2	0.86	1.84	2.7033 (19)	176
N2—H2*A*···O3^iii^	0.86	2.00	2.816 (3)	158
N2—H2*B*···O3	0.86	1.99	2.830 (2)	165
O4—H4···O2	0.82	1.81	2.534 (2)	147
C3—H3···O1^i^	0.93	2.48	3.374 (3)	160

Symmetry codes: (i) −*x*+3, −*y*+1, −*z*+2; (iii) −*x*+1, −*y*+1, −*z*+1.
